# Avascular necrosis less frequently found in systemic lupus erythematosus patients with the use of alternate day corticosteroid

**DOI:** 10.3906/sag-1908-182

**Published:** 2020-02-13

**Authors:** İsmail DOGAN, Umut KALYONCU, Levent KILIÇ, Ali AKDOĞAN, Ömer KARADAĞ, Sedat KİRAZ, Şule A. BİLGEN, İhsan ERTENLİ

**Affiliations:** 1 Division of Rheumatology, Department of Internal Medicine, Yıldırım Beyazıt University, Ankara Turkey; 2 Division of Rheumatology, Department of Internal Medicine, Hacettepe University, Ankara Turkey

**Keywords:** Avascular necrosis, alternate day steroid usage, systemic lupus erythematosus

## Abstract

**Background/aim:**

Avascular necrosis (AVN) is the death of bone due to compromise of blood flow. The etiology of AVN is multifactorial; corticosteroid usage is the second most significant factor after trauma, and systemic lupus erythematosus (SLE) is the most common underlying disease. The objective of this study was to assess the factors of AVN in SLE patients.

**Materials and methods:**

The study included 127 patients with SLE who fulfilled 1997 American College of Rheumatology (ACR) revised criteria. Demographic data, age at SLE diagnosis, disease duration, disease activity, body mass index, clinical findings, antiphospholipid syndrome, steroid usage, dose and duration, comorbid diseases, and smoking history were recorded.

**Results:**

AVN was found in 11 of 127 (8.7%) SLE patients. Hyperlipidemia (P < 0.001), cushingoid body habitus (P < 0.001), and proteinuria (P = 0.013) were found at higher rates in the AVN group. All of the 11 AVN cases had osteoporosis (P < 0.02). In multivariate regression analysis, daily steroid usage was the only factor for development of AVN in SLE.

**Conclusion:**

The hypothesis of our study was that an alternate day steroid regimen may decrease AVN frequency in SLE patients.

## 1. Introduction

Avascular necrosis (AVN) is bone death due to compromise of blood flow that leads to arthralgia, bone destruction, and loss of function in the joint. AVN etiology is multifactorial, and steroid usage is the second most important factor after trauma. Systemic lupus erythematosus (SLE) is the most common underlying disease in AVN [1]. In 1960, Dubois and Cozen reported AVN in SLE patients [2]. The prevalence of AVN in SLE patients is around 3%–52%, according to diagnostic methods and evaluation of symptomatic or asymptomatic patients [3]. The risk factors for AVN in SLE patients were shown to be young age, sex, cushingoid body habitus, Raynaud’s phenomenon, thrombophlebitis, vasculitis, nephritis, cerebritis, interstitial pneumonitis, pleural effusion, antiphospholipid syndrome, preeclampsia, hypertension, anemia, thrombocytopenic thrombotic purpura, smoking, and migraine [4]. The objective of this study was to evaluate associated factors for the development of AVN in our SLE cohort.

## 2. Materials and methods

A total of 127 consecutive SLE patients (86.6% female) were enrolled in this cross-sectional study during 10 months in our outpatient clinic. Ethical approval was received from the local ethical committee. Inclusion criteria of this study were being ≥18 years old and fulfillment of the 1997 American College of Rheumatology (ACR) revised criteria for the classification of SLE [5]. 

Demographic data, clinical, and laboratory features were recorded from a standard questionnaire and from the patients’ files. Potential associated factors of AVN such as sex, age, body mass index, age at disease diagnosis, disease duration, smoking history, hypertension, diabetes mellitus, coronary artery disease, hyperlipidemia, anemia, osteoporosis, menopause, SLE disease activity score, antiphospholipid antibodies positivity, and corticosteroid usage were noted. 

SLE disease activity index score (SLEDAI-2K) [6] was recorded for assessment of disease activity. SLEDAI scores were also calculated according to the situation 6 months prior to AVN diagnosis.

Cases with antiphospholipid syndrome were defined according to the 1999 classification criteria [7]. Lupus anticoagulant positivity and/or anticardiolipin antibody positivity were recorded one by one separately. Anti-beta 2 glycoprotein I antibodies could not be evaluated.

The corticosteroid usage of the patients was analyzed very carefully, from SLE diagnosis to introduction to the study of all the patients individually. The duration and dosage of the corticosteroid therapy, daily or alternate day regimen, and intravenous pulse steroid usage were recorded separately. In addition to total steroid dose, total daily or alternate day, total pulse steroid doses, and mean daily steroid doses were calculated. High-dose steroid was defined as >30 mg/day steroid usage at any time [8]. In our clinic, we prefer daily and pulse steroid use with induction treatment; and we prefer alternate day steroid use in the maintenance treatment. 

AVN was diagnosed according to routine clinical practice, in symptomatic patients by plain X-ray and/or magnetic resonance imaging (MRI). 

### 2.1. Statistical analysis

Data analysis was made by SPSS 15.0 (SPSS Inc., Chicago, IL, USA) and LogXact packet program, and P < 0.05 was considered as statistical significance. Descriptive statistics were given as mean ± standard deviation for numerical data and as number and percent for qualitative data. To compare numerical data between the AVN developed group (AVN group) and the AVN nondeveloped group (non-AVN group), an independent t-test was used; to compare percentile data, chi-square test and Fischer’s exact test were applied. Independent variables that were significant after evaluation with single variable tests were evaluated by single variable logistic regression and exact logistic regression analysis to determine their odds ratio. To determine significant variables’ effects on AVN development, multiple logistic regression analysis was applied. 

## 3. Results

### 3.1. Demographic and clinical features

AVN was found in 11 of 127 (8.7%) SLE patients. Affected AVN sides were the femoral head (73%), shoulder (27%), knee (27%), and metatarsal head (9%). Multiple site involvement was found in 5patients, and 24 sites were affected in total. All AVN patients had osteoporosis and were under bisphosphonates, calcium, and vitamin D therapy. 

Demographic features, diagnostic criteria, and comorbid factors did not differ between the AVN group and the non-AVN group; hyperlipidemia, however, was significantly higher in the AVN group (P < 0.001). Antiphospholipid syndrome, Raynaud’s phenomenon, vasculitis, and SLEDAI, which were evaluated greatly in previous studies, were the same in both groups**.** However, cushingoid body habitus, proteinuria, and osteoporosis were significantly higher in the AVN group (P < 0.001, P < 0.013, and P < 0.03, respectively) (Table 1).

**Table 1:** Demographic and laboratory features of patients with and without AVN.

	AVN (+) n = 11	AVN (-) n = 116	P-value
Female, n (%)	9 (82)	101 (87)	0.6
Age (mean ± SD)	34.5 (10.6)	39.1 (14.2)	0.3
Age at SLE diagnosis (mean ± SD)	28.5 (11.4)	32.3 (14.2)	0.4
Disease duration months (mean ± SD)	82.6 (74.3)	88.7 (67.5)	0.8
Malar rash, n (%)	6 (54.5)	54 (46.6)	0.8
Photosensitivity, n (%)	8 (72.7)	71 (61.2)	0.5
Oral ulcer, n (%)	1 (9.1)	31 (26.7)	0.3
Discoid rash, n (%)	0 (0)	12 (10.3)	0.6
Arthritis, n (%)	9 (81.8)	89 (76.7)	1.0
Neurologic involvement, n (%)	2 (18.2)	18 (15.5)	0.7
Renal involvement, n (%)	7 (63.6)	49 (42.2)	0.2
Serositis, n (%)	2 (18.2)	25 (21.6)	1.0
Hematologic involvement, n (%)	5 (45.5)	54 (46.6)	1.0
Raynaud’s phenomenon, n (%)	7 (6.6)	40 (36.0)	0.1
Vasculitis, n (%)	1 (9.1)	18 (15.7)	1.0
ANA, n (%)	11 (100)	114 (98.3)	1.0
ACA, n (%)	4 (36.4)	26 (22.4)	0.3
LA, n (%)	3 (27.3)	20 (17.4)	0.4
APL carriers, n (%)	6 (54.5)	31 (26.7)	0.1
APLS, n (%)	5 (45.5)	17 (54.8)	0.4
Persistent proteinuria ≥ 500 mg/day, n (%)	4 (36.4)	12 (10.3)	0.013
Anemia, n (%)	6 (54.5)	75 (64.7)	0.5
SLEDAI -2K (mean ± SD)	5.3 (3.5)	4.0 (4.8)	0.9
Comorbid diseases, n (%)HypertensionDiabetes mellitusCoronary arterial diseaseHyperlipidemiaBMI ≥ 25 Smoking	5 (45.5)0 (0)0 (0)4 (36.4)7 (63.6)4 (36.4)	27 (23.3)11 (9.5)4 (3.4)11 (9.5)57 (49.1)29 (25.0)	0.060.61.0<0.0010.530.47
Cushingoid body habitus, n (%)	8 (72.7)	28 (24.3)	<0.001
Osteoporosis, n (%)	11 (100)	68 (66.7)	0.03

ACA: Anticardiolipin antibody positivity, ANA: Antinuclear antibody, APL carriers: antiphospholipid antibody carriers, APLS: Cases whom have both anticardiolipin and/or lupus anticoagulant positivity and abortus, thrombosis clinic, AVN: Avascular necrosis, BMI: body mass index, GFR: glomerular filtration rate, LA: Lupus anticoagulant positivity.

### 3.2. Corticosteroid therapy regimen

Corticosteroid was used by 122 of 127 (96.1%) SLE patients during the treatment protocol (Table 2). All AVN group patients had taken high-dose steroid therapy at some time in their treatment duration. There was no difference in corticosteroid usage ratio, corticosteroid usage duration, total corticosteroid dosage, or total corticosteroid dose/corticosteroid duration between patients with and without AVN. However, daily corticosteroid usage, high-dose steroid usage, and total daily steroid dosage were significantly higher in patients with AVN than in those without AVN. Patients without AVN had used alternate day corticosteroid more frequently. 

**Table 2:** Steroid therapy method, dose, duration, and form in patients with and without AVN.

	AVN (+), n = 11	AVN (-), n = 116	P-value
Corticosteroid usage, n (%)	11 (100)	111 (95.7)	0.7
Alternate day corticosteroid usage, n (%)	5 (45.5)	101 (91.0)	0.003
Daily corticosteroid usage, n (%)	11 (100)	54 (48.6)	0.001
Pulse corticosteroid usage, n (%)	4 (36.4)	42 (37.8)	1.0
High dose corticosteroid usage, n (%)	11 (100)	78 (70.3)	0.035
Corticosteroid duration (month) (mean ± SD)	75.1 (71.5)	81.6 (68.0)	0.9
Total corticosteroid dosage (gr) (mean ± SD)	19.6 (14.6)	21.4 (20.6)	0.8
Total corticosteroid dosage (mg) /corticosteroid duration (day) = daily dosage (mean ± SD)	12.0 (7.5)	15.7 (22.5)	0.8

High-dose steroid: >30 mg/day steroid usage in any period of therapy; total steroid dose: total steroid dose used throughout therapy duration; steroid duration: steroid therapy duration as month. NA: Standard errors could not be estimated in exact logistic regression analysis. Inf: Infinitive. AVN: avascular necrosis, SLE: systemic lupus erythematosus

Based on the results of our study, hyperlipidemia, osteoporosis, daily corticosteroid usage, total daily corticosteroid dose, high dose corticosteroid therapy, presence of proteinuria, and cushingoid body habitus were statistically significant factors for the development of AVN. According to the multivariate logistic regression analysis, daily steroid usage was associated independently with AVN occurrence (Table 3).

**Table 3:** Multivariate logistic regression analysis of predictive factors of AVN in SLE.

	Beta	Standard error	P-value	Odds ratio (95% confidence interval)
Hyperlipidemia	1.549	0.973	0.237	4.71 (0.489–63.94)
Osteoporosis	0.379	NA	0.765	1.46 (0.156–inf)
Daily steroid usage	2.664	NA	0.014	14.35 (1.645–inf)
Total daily steroid dosage (gr)	-0.036	0.036	0.297	0.964 (0.889–1.03)
Persistent proteinuria ≥ 500 mg/day	0.520	0.865	0.836	1.682 (0.194–14.19)
High dose steroid	1.324	NA	0.251	3.758 (0.440–inf)
Cushingoid body habitus	0.452	0.855	0.916	1.571 (0.215–13.57)

## 4. Discussion

In our study, hyperlipidemia, osteoporosis, daily corticosteroid usage, total daily corticosteroid dosage, persistent proteinuria, high-dose corticosteroid, and cushingoid body habitus variables were found to be individual risk factors for the development of AVN. However, multivariate analysis showed that only daily corticosteroid usage was a unique factor for AVN. From another point of view, alternate day corticosteroid usage was the protective factor for the development of AVN. Interestingly, total corticosteroid dosage and corticosteroid duration did not influence the development of AVN in this study. 

The relationship between corticosteroid usage and AVN was first reported in renal transplant patients [9]. Thereafter, physicians noticed the relation between AVN and SLE. In a large cohort of 1729 SLE patients with symptomatic AVN, with more than 40 years of follow-up, multivariate analysis had shown that only corticosteroids were a primary risk for AVN [10]. Its prevalence changed from 3% to 52% between symptomatic patients and asymptomatic SLE patients [3]. AVN prevalence was reported as 6% in a study from Turkey with 868 SLE cases [11], the ratio of which was similar to our study (8.7%). In this study, we only assessed symptomatic patients; thus we can only say “at least” or “symptomatic” prevalence of AVN in SLE patients. 

Various conflict risk factors were defined in the development of AVN in SLE patients. Some reports stated that young age is a risk factor for AVN occurrence [12–14]. Indeed, our patients with AVN were younger than those without AVN; however, the difference was not statistically significant. Hypertension, anemia, male sex, and diabetes mellitus were the other potential risk factors in the literature, but we did not find this kind of relationship [11,15]. In those risk factors, hyperlipidemia is an important risk factor for AVN, and some studies suggested that antihyperlipidemic therapy may decrease AVN risk [16,17]. In a pathogenetic view, corticosteroid treatment may increase adipogenesis and fat hypertrophy in bone marrow, and cause intraosseous pressure increase, interrupt blood flow to the femoral head, and result in ischemic AVN [18]. In the present study, we found that hyperlipidemia was more frequent in the AVN group, but multivariate logistic regression analysis did not show a strong relation between hyperlipidemia and AVN. Cozen and Zhang et al. reported that AVN was frequent in active, severe lupus patients [15,19]. However, it is well known that severe organ involvement is associated with high-dosage corticosteroid usage, and we did not demonstrate this connection. Antiphospholipid syndrome is another risk factor for AVN that was investigated by most studies with conflicting results [20–23]. Our study has not suggested this relation. Osteoporosis, a frequent complication of corticosteroid and SLE, was found in all of our AVN cases. All of them were also receiving therapy for osteoporosis. High-dose corticosteroid may decrease bone formation rapidly and permanently, and increase bone resorption rapidly and temporarily [24]. Indeed, corticosteroid induced osteoblast and osteocyte apoptosis may be the common mechanisms for osteoporosis and AVN [25].

There was a strong association with daily corticosteroid usage and AVN in our study. In 10 of a total of 16 reports, it was suggested that mean daily, maximum daily, or high corticosteroid dosage was related with AVN occurrence. Eight of 13 reports concerned with cumulative corticosteroid dosage showed its relation with AVN [26]. The main result of our study was the superior effect for the protection of AVN by usage of alternate day corticosteroid. In a study that had compared long-term daily and alternate day corticosteroid, although cumulative corticosteroid dosage was higher in the alternate day group, corticosteroid side effects such as moon face, psychiatric complications, and regression in growth were low in the alternate day group; furthermore, similar remission rates were obtained for both groups [27]. In an animal study, AVN prevalence was 45% versus 8% between the daily corticosteroid group and intermittent corticosteroid group [28]. Although cumulative corticosteroid exposure was similar in both groups, serum cortisol levels of the daily steroid group were fully suppressed, but the intermittent group had partial adrenal recovery. It was interpreted that partial recovery of adrenal suppression in the intermittent steroid group was the factor for low AVN risk. In ALL and renal transplant patients, low AVN prevalence and low infection risk were reported with alternate day corticosteroid [29,30]. Due to the acceptable side effect profile and similar clinical outcome of alternate day corticosteroid with daily corticosteroid, we have routinely preferred alternate day corticosteroid for long-term maintenance corticosteroid therapy in our outpatient clinic for more than 20 years. 

The main limitation was the design of the study. We assessed SLE patients cross-sectionally; patient data was recorded from patients and also from medical files. We could not track patients prospectively, therefore, some of the patients who had AVN and joint prosthesis may be withdrawn from our follow-up. Hence, our AVN prevalence should be accepted as an “at least” frequency. We assessed only symptomatic patients through imaging. Thus, it is possible to find a high prevalence when evaluating all patients regularly by X-ray or MRI. Lastly, the number of AVN cases may not be enough for an absolute conclusion about possible AVN risk factors. 

In conclusion, to decrease AVN risk in suitable patients, alternate day steroid usage may be encouraged in patients who are candidates for long-term maintenance corticosteroid usage. However, physicians should keep in mind that alternate day corticosteroid usage is also closely related with other appropriate immunosuppressive therapies. 
